# PPP2R5B regulates ANPEP expression and TGEV entry via dephosphorylation of HSF1 at Ser304/Ser308

**DOI:** 10.1128/jvi.00166-26

**Published:** 2026-06-11

**Authors:** Yichen Yang, Zhelin Su, Xiyue Zhang, Yubei Tan, Hakimeh Baghaei Daemi, Zhen Fu, Meijie Gao, Shengsong Xie, Limeng Sun, Guiqing Peng

**Affiliations:** 1 State Key Laboratory of Agricultural Microbiology, College of Veterinary Medicine, Huazhong Agricultural University 627716, Wuhan, China; 2 Key Laboratory of Preventive Veterinary Medicine in Hubei Province, the Cooperative Innovation Center for Sustainable Pig Production, Wuhan, China; 3 Key Laboratory of Agricultural Animal Genetics, Breeding and Reproduction of Ministry of Education & Key Lab of Swine Genetics and Breeding of Ministry of Agriculture and Rural Affairs, Huazhong Agricultural University 47895, Wuhan, China; 4 Key Laboratory of Prevention & Control for African Swine Fever and Other Major Pig Diseases, Ministry of Agriculture and Rural Affairs, Wuhan, China; 5 Hubei Hongshan Laboratory, Frontiers Science Center for Animal Breeding and Sustainable Production, Wuhan, China; The Ohio State University, Columbus, Ohio, USA

**Keywords:** PPP2R5B, HSF1, ANPEP, coronavirus entry, dephosphorylation

## Abstract

Transmissible gastroenteritis virus (TGEV) is an enteric coronavirus responsible for severe gastrointestinal disease in swine; however, the host determinants and mechanisms underlying viral entry remain poorly defined. This study systematically examines the role of the serine/threonine protein phosphatase 2A (PP2A) regulatory subunit B'β (PPP2R5B) during TGEV infection. Genetic ablation of PPP2R5B in PK-15 cells significantly reduced TGEV infectivity. Stage-specific analyses revealed that loss of PPP2R5B selectively impaired viral entry, while viral replication and release were unaffected. Transcriptomic profiling and functional validation identified the TGEV receptor aminopeptidase N (ANPEP) as a critical downstream effector of PPP2R5B. PPP2R5B-mediated regulation of ANPEP expression was independent of the AMPK, PI3K/AKT, ERK, and WNT/β-catenin signaling pathways. Instead, PPP2R5B directly interacted with heat shock factor 1 (HSF1) and promoted dephosphorylation of HSF1 at Ser304 and Ser308, thereby reducing HSF1-dependent transcriptional activation of ANPEP. These findings reveal a previously unrecognized PPP2R5B–HSF1–ANPEP regulatory axis that governs TGEV cellular entry and suggest that host dephosphorylation-dependent regulation may represent a potential target for antiviral intervention.

**IMPORTANCE:**

Coronavirus entry is a critical determinant of viral tropism and pathogenicity. This study identifies PPP2R5B, a regulatory subunit of the PP2A phosphatase complex, as a host factor that facilitates TGEV entry by controlling the expression of the cellular receptor ANPEP. We demonstrate that PPP2R5B regulates ANPEP transcription through modulation of HSF1 phosphorylation, independent of several canonical signaling pathways. These findings uncover a host phosphatase-dependent regulatory mechanism underlying coronavirus entry and highlight host signaling components as potential targets for antiviral intervention.

## INTRODUCTION

Coronaviruses (CoVs) are a family of enveloped, positive-sense, single-stranded RNA viruses that can cause respiratory and intestinal diseases in both humans and various animals. They are classified into four genera: α, β, γ, and δ (1, 2). In addition, porcine transmissible gastroenteritis virus (TGEV), which belongs to the alphacoronavirus genus, can induce acute enteritis in pigs of all ages, with a mortality rate of up to 100% in piglets within 2 weeks of birth (3). Although significant progress has been made in TGEV research over the past few decades, numerous challenges remain regarding viral virulence and cross-species transmission. Notably, highly virulent recombinant TGEV strains continue to be observed due to frequent evolutionary and recombination events (4, 5). Meanwhile, epidemiological findings indicate intricate connections between TGEV and newly identified swine enteric coronaviruses (SeCoV) (6, 7), canine-feline coronaviruses (CCoV-HuPn-2018) (8), as well as feline coronaviruses (FCoV) (9), collectively posing a substantial threat to public health security.

Mammals orchestrate gene expression through complex regulatory mechanisms, prominently involving post-translational modifications (PTMs). Phosphorylation, one of the most prevalent and extensively studied PTMs, plays a central role in nearly all cellular biological processes. Protein phosphorylation participates in regulating signal transduction, cell cycle progression, metabolic reprogramming, and gene transcription, collectively influencing cell growth, differentiation, apoptosis, and stress responses (10, 11). Notably, substantial evidence indicates that protein phosphorylation is closely associated with pathogen invasion, replication, immune evasion, and pathogenicity (12, 13).

PPP2R5B encodes the B56β regulatory subunit of protein phosphatase 2A (PP2A), a serine/threonine phosphatase, and serves as an essential part of the PP2A holoenzyme. As a major member of the B56 regulatory subunit family, this protein dictates both the substrate specificity and subcellular localization of the PP2A enzyme (14). It has been shown to play important roles in insulin signaling (15) as well as in cell division and differentiation (16, 17). Of note, many components of intracellular signaling cascades act as direct or indirect substrates of PP2A complexes that contain PPP2R5B, thereby allowing PPP2R5B to participate in canonical pathways such as PI3K/AKT (18, 19) and Ras/ERK (20). Even so, additional downstream substrates and signaling mechanisms regulated by PPP2R5B have yet to be identified. Furthermore, the functional involvement of PPP2R5B in virus–host interactions and viral infection remains poorly understood.

Heat shock factor 1 (HSF1) serves as a master transcriptional regulator of heat shock response genes, including molecular chaperones, such as heat shock proteins (HSPs), which are induced in response to proteotoxic stress. The activity of HSF1 is tightly controlled by post-translational modifications, particularly phosphorylation. Phosphorylation at residues S230 (21), S320 (22), S326 (23), and S419 (24) enhances the transcriptional capacity of HSF1, whereas phosphorylation at Ser303 and Ser307 suppresses its transactivation potential (25).

This study identified PPP2R5B as a key host factor regulating TGEV entry. Knockout of PPP2R5B using CRISPR/Cas9 significantly suppressed TGEV infection and specifically blocked the viral entry phase. Further mechanistic analysis revealed that PPP2R5B deficiency acts independently of the canonical signaling pathways examined here; instead, it leads to increased phosphorylation of HSF1 at Ser304 and Ser308, which correlates with reduced transcription of ANPEP, a known functional receptor for TGEV. Together, these results uncover a previously undescribed PPP2R5B–HSF1–ANPEP regulatory axis that controls TGEV entry and deepen our understanding of host dephosphorylation-dependent mechanisms in coronavirus infection.

## RESULTS

### PPP2R5B is a host factor required for TGEV infection

**Fig 1:**
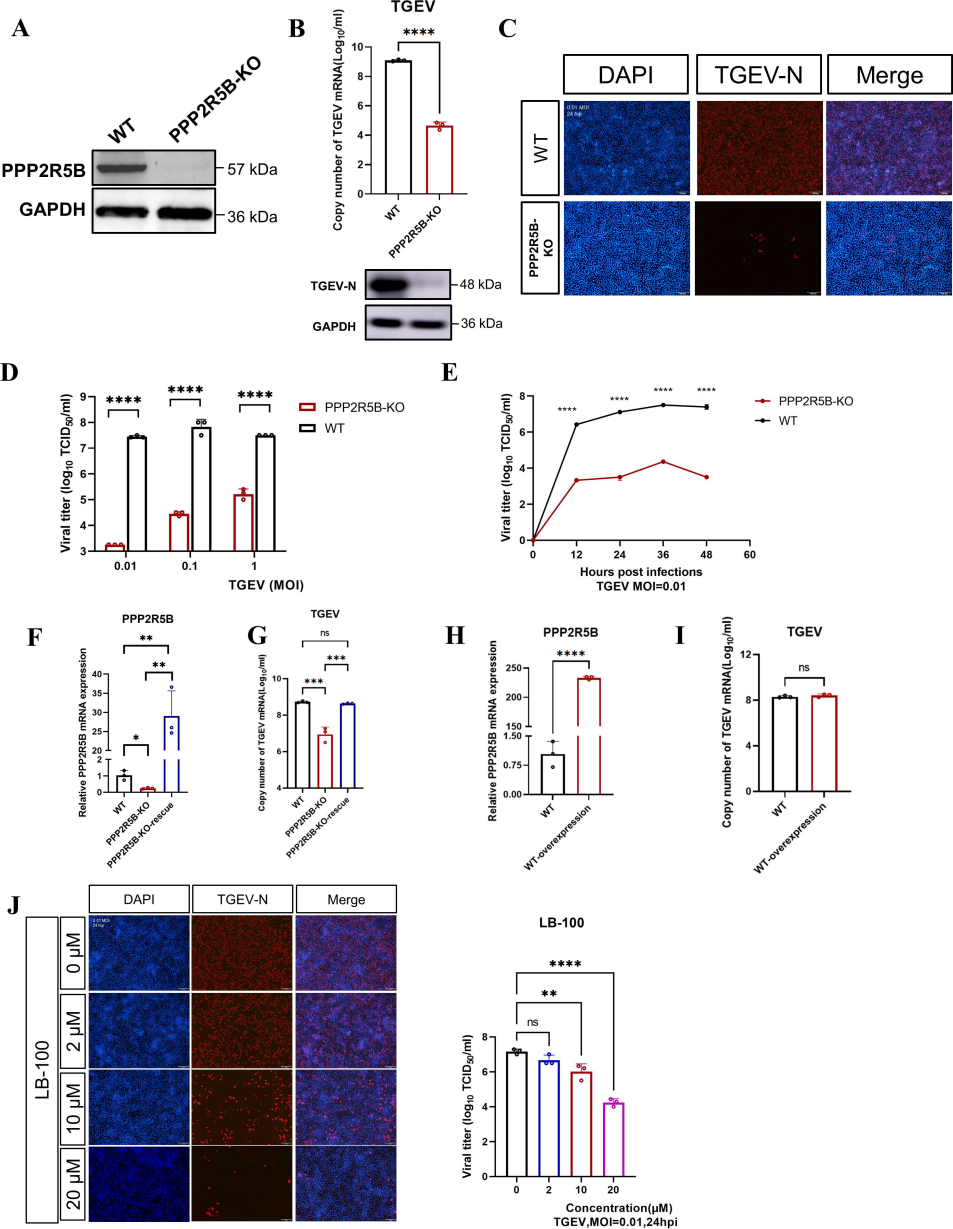
PPP2R5B is a host factor required for TGEV replication. (**A**) PPP2R5B protein levels in WT and PPP2R5B KO cells were analyzed by Western blot. (**B and C**) TGEV N mRNA (**B**) and protein (**C**) levels in WT and PPP2R5B KO cells after infection (MOI = 0.01). (**D**) PPP2R5B KO and WT cells were infected with TGEV at different MOIs (0.01, 0.1, and 1). (**E**) Multi-step growth kinetics of TGEV in WT and PPP2R5B KO cells. (**F and G**) PPP2R5B expression (**F**) and TGEV N gene levels (**G**) in WT, PPP2R5B KO, and PPP2R5B-rescued cells. (**H and I**) PPP2R5B expression (**H**) and TGEV N gene levels (**I**) in control and PPP2R5B-overexpressing cells after infection (MOI = 0.01). (**J**) Effect of PP2A inhibition on TGEV infection, assessed by immunofluorescence and viral titration. Data are representative of at least three independent experiments. Statistical significance was determined by Student’s *t*-test or ANOVA as indicated. *P < 0.05; **P < 0.01; ***P < 0.001; ****P < 0.0001.

To investigate the role of PPP2R5B during TGEV infection, a clonal PPP2R5B knockout (KO) PK-15 cell line was generated using the CRISPR/Cas9 gene-editing system. Sanger sequencing confirmed a single-base insertion in PPP2R5B KO cells (Fig. S1A), and ablation of PPP2R5B protein expression was validated by Western blot analysis (Fig. 1A). No significant difference in cell proliferation was observed between wild-type (WT) and PPP2R5B KO cells, as assessed by an MTS assay (Fig. S1B).

Viral mRNA and protein levels were subsequently evaluated in WT and PPP2R5B KO cells infected with TGEV at a multiplicity of infection (MOI) of 0.01 using RT-qPCR and Western blot analysis, respectively (Fig. 1B). Both TGEV N mRNA and protein levels were significantly reduced in PPP2R5B KO cells compared with WT cells at 24 h post-infection (hpi). Consistent with these results, indirect immunofluorescence analysis revealed markedly reduced TGEV N protein expression in PPP2R5B KO cells (Fig. 1C). In addition, viral titers were significantly lower in PPP2R5B KO cells than in WT cells across multiple MOIs (0.01, 0.1, and 1) (Fig. 1D) and at various time points (12, 24, 36, and 48 hpi) (Fig. 1E). Collectively, these data demonstrate that PPP2R5B knockout markedly impairs TGEV infection.

To further assess the requirement of PPP2R5B for TGEV infection, a PPP2R5B-KO rescue cell line was generated via lentivirus-mediated transduction (Fig. 1F). TGEV replication was restored to levels comparable to those in WT cells in PPP2R5B-KO rescue cells (Fig. 1G). In parallel, a PPP2R5B-overexpressing cell line was generated to evaluate the effect of PPP2R5B overexpression (Fig. 1H). As shown in Fig. 1I, overexpression of PPP2R5B did not significantly alter TGEV infection.

To determine whether PPP2R5B promotes TGEV infection through its role within the PP2A holoenzyme, LB-100, a selective inhibitor of PP2A phosphatase activity, was used (26). Treatment with LB-100 at non-cytotoxic concentrations (Fig. S1C) resulted in a significant dose-dependent inhibition of TGEV infection, as evidenced by both indirect immunofluorescence and viral titer assays (Fig. 1J). These results establish that PPP2R5B (PP2A B56β) promotes TGEV infection through its specific function as a regulatory subunit within the PP2A holoenzyme complex.

### PPP2R5B knockout suppresses the entry stage of TGEV infection

**Fig 2:**
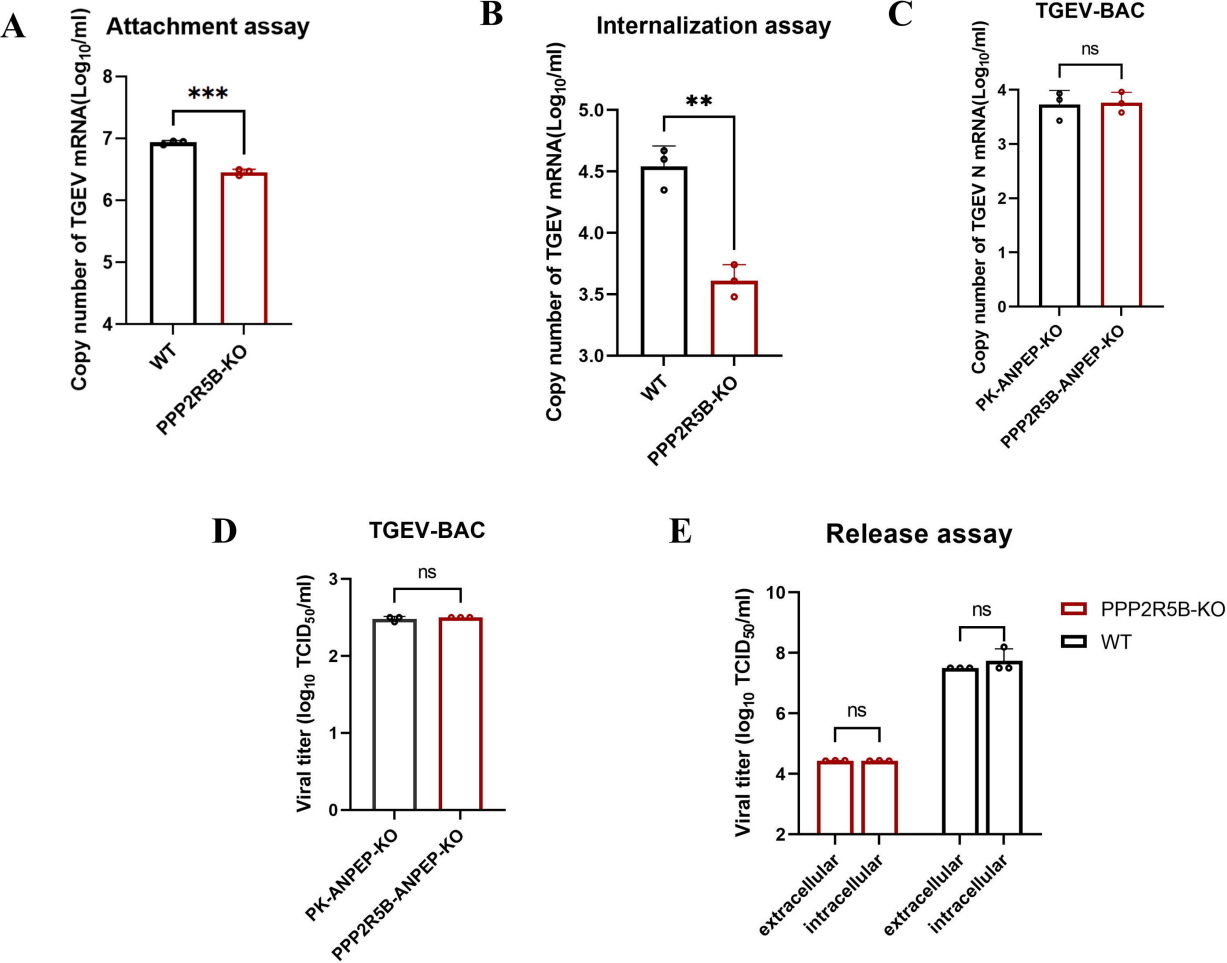
PPP2R5B knockout suppresses the entry stage of TGEV infection. (**A**) Viral attachment to WT and PPP2R5B KO cells, quantified by cell-associated viral RNA. (**B**) Viral internalization in WT and PPP2R5B KO cells, measured after removal of surface-bound virus. (**C and D**) TGEV replication at 60 h post-transfection of TGEV-BAC in ANPEP KO and PPP2R5B/ANPEP double KO cells, assessed by viral RNA levels (**C**) and titers (**D**). (**E**) Intracellular and extracellular viral titers in WT and PPP2R5B KO cells. Data are representative of at least three independent experiments. *P* < 0.05; **P < 0.01; ***P < 0.001; ****P < 0.0001. *P*-values were determined by two-tailed unpaired Student’s *t*-tests.

Virus attachment and internalization assays were performed to assess whether PPP2R5B knockout affects viral attachment and entry during the TGEV infection cycle. PPP2R5B KO and WT cells were incubated with TGEV at 4°C for 1 h to permit viral attachment. In the internalization assay, surface-bound virions were removed by washing with acidic PBS (pH 3.0). RT-qPCR analysis demonstrated a significant reduction in viral internalization efficiency in PPP2R5B KO cells compared with WT cells (Fig. 2A and B).

To determine whether PPP2R5B influences viral replication, ANPEP KO PK-15 cells and PPP2R5B–ANPEP double-KO cells were generated and transfected with TGEV BAC plasmids to bypass ANPEP-mediated viral entry. Both RT-qPCR (Fig. 2C) and TCID₅₀ assays (Fig. 2D) indicated that deletion of PPP2R5B did not affect viral replication.

Viral release assays showed that both extracellular and intracellular viral titers were similarly reduced in PPP2R5B KO cells compared with WT cells (Fig. 2E), indicating that PPP2R5B deficiency does not impair TGEV release.

### PPP2R5B knockout inhibits TGEV entry by downregulating ANPEP expression

**Fig 3:**
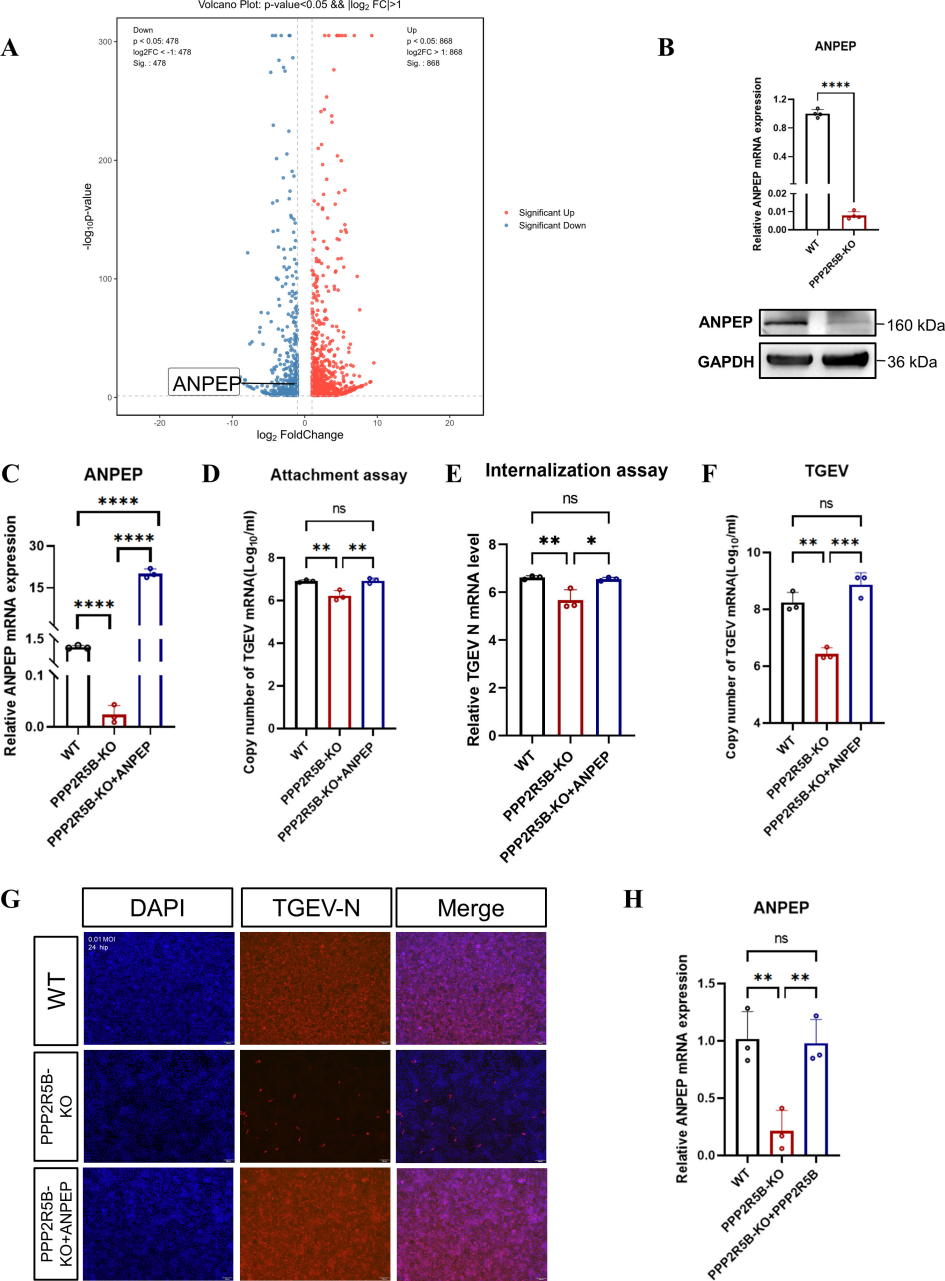
PPP2R5B regulates TGEV entry through ANPEP. (**A**) Volcano plot of differentially expressed genes in WT and PPP2R5B KO PK-15 cells. (**B**) ANPEP mRNA and protein levels in WT and PPP2R5B KO cells. (**C**) ANPEP mRNA expression in WT, PPP2R5B KO, and ANPEP overexpressing PPP2R5B KO cells. (**D and E**) Viral attachment (**D**) and internalization (**E**) in WT, PPP2R5B KO, and ANPEP-overexpressing PPP2R5B KO cells. (**F and G**) TGEV N mRNA (**F**) and protein (**G**) levels in WT, PPP2R5B KO, and ANPEP-overexpressing PPP2R5B KO cells following TGEV infection (MOI = 0.01). (**H**) ANPEP mRNA expression in WT, PPP2R5B KO, and PPP2R5B-rescued cells. Data are representative of at least three independent experiments. Statistical significance was determined by Student’s *t*-test or ANOVA as indicated. *P < 0.05; **P < 0.01; ***P < 0.001; ****P < 0.0001.

RNA sequencing (RNA-seq) was performed on WT and PPP2R5B KO cells to elucidate the mechanism by which PPP2R5B regulates TGEV entry. Transcriptomic analysis revealed that ANPEP expression was significantly downregulated in PPP2R5B KO cells (Fig. 3A). This observation was further validated at both the mRNA and protein levels by RT-qPCR and Western blot analyses (Fig. 3B).

To further define the functional relationship between PPP2R5B and ANPEP, a PPP2R5B KO + ANPEP rescue cell line was generated by lentivirus-mediated transduction (Fig. 3C). Viral attachment (Fig. 3D) and internalization assays (Fig. 3E) showed that both viral attachment and entry were restored to WT levels in the rescue cells. Subsequent infection assays indicated that TGEV N mRNA levels in PPP2R5B KO + ANPEP cells were comparable to those in WT cells (Fig. 3F). Indirect immunofluorescence assays further confirmed restoration of TGEV N protein expression (Fig. 3G). Meanwhile, ANPEP expression was also effectively restored upon PPP2R5B restoration (Fig. 3H). Collectively, these results indicate that loss of PPP2R5B impairs TGEV entry by downregulating ANPEP expression.

### PPP2R5B modulates ANPEP expression independently of the AMPK, PI3K/AKT, ERK, and WNT signaling pathways

**Fig 4:**
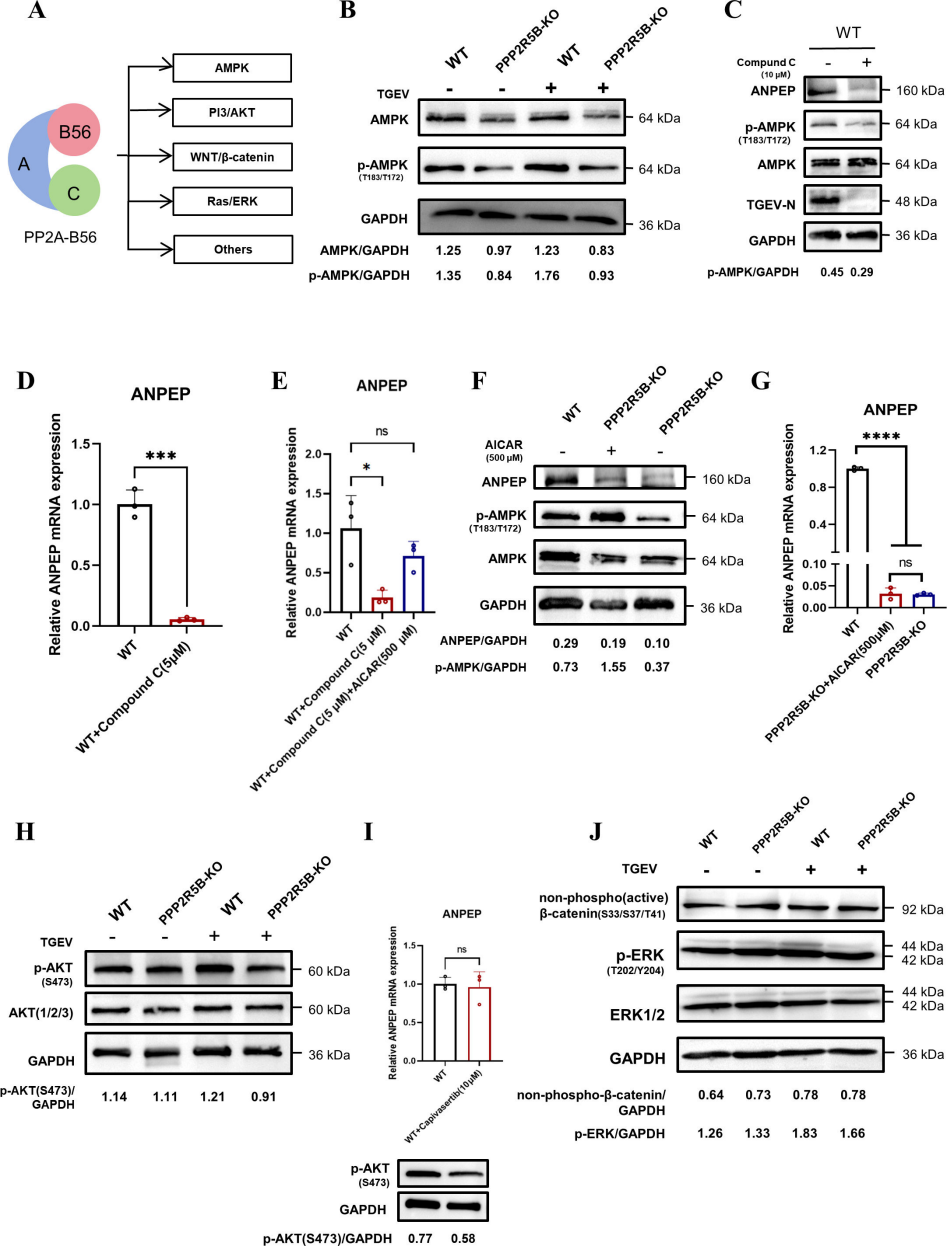
PPP2R5B modulates ANPEP expression independently of the AMPK, PI3K/AKT, ERK, and WNT signaling pathways. (**A**) Schematic overview of candidate signaling pathways downstream of PP2A-B56β. (**B**) AMPK phosphorylation and expression in WT and PPP2R5B KO cells under TGEV infection or mock conditions. (**C and D**) Effects of AMPK inhibition (Compound C, 5 μM, 24 h) on p-AMPK, AMPK, ANPEP, and TGEV-N protein levels (**C**) and ANPEP mRNA levels (**D**) in WT cells. (**E**) ANPEP mRNA levels in WT cells following treatment with Compound C (5 μM, 30 h) or Compound C pretreatment (5 μM, 18 h), followed by AICAR (500 μM, 12 h). (**F and G**) p-AMPK, AMPK, and ANPEP protein levels (**F**) and ANPEP mRNA levels (**G**) in WT, PPP2R5B KO, and PPP2R5B KO cells treated with AICAR (500 μM, 24 h). (**H**) AKT phosphorylation and total protein levels in WT and PPP2R5B KO cells under TGEV infection or mock conditions. (**I**) Effects of AKT inhibition (Capivasertib, 10 μM, 24 h) on AKT phosphorylation and ANPEP mRNA levels in WT cells. (**J**) β-catenin and ERK activity in WT and PPP2R5B KO cells under TGEV infection or mock conditions. Data are representative of at least three independent experiments. Statistical significance was determined by Student’s *t*-test or ANOVA as indicated. *P < 0.05; **P < 0.01; ***P < 0.001; ****P < 0.0001.

The PP2A-B56β holoenzyme has been reported to modulate multiple downstream signaling pathways, including AMPK (27), AKT (18, 19), ERK1/2 (20), and WNT/β-catenin (28) (Fig. 4A), which have been implicated in the regulation of coronavirus receptor expression. To systematically assess the contribution of these pathways, a series of validation experiments was performed.

Western blot analysis showed that both total AMPK expression and AMPK phosphorylation were significantly reduced in PPP2R5B KO cells (Fig. 4B). To determine whether AMPK activity contributes to ANPEP transcription, WT cells were treated with the AMPK inhibitor Compound C. Treatment with Compound C (5 μM) markedly suppressed AMPK phosphorylation and reduced ANPEP mRNA and protein expression (Fig. 4C and D). Subsequent treatment with the AMPK activator AICAR partially restored ANPEP transcription in WT cells pretreated with Compound C (Fig. 4E). In contrast, although AICAR treatment restored AMPK phosphorylation in PPP2R5B KO cells, ANPEP transcription remained unchanged (Fig. 4F and G).

The effect of PPP2R5B on AKT signaling was examined by analyzing phosphorylation of AKT at Ser473 and Thr308. Both phosphorylation sites were significantly reduced in PPP2R5B KO cells (Fig. 4H). However, pharmacological inhibition of AKT phosphorylation with capivasertib did not affect ANPEP mRNA expression (Fig. 4I).

Western blot analysis showed no significant differences in the levels of active β-catenin or phosphorylated ERK1/2 (Thr202/Tyr204) between PPP2R5B KO and WT cells (Fig. 4J), suggesting that WNT/β-catenin and ERK1/2 signaling are not affected by PPP2R5B loss.

Collectively, these data indicate that AMPK, AKT, ERK1/2, and WNT/β-catenin signaling pathways are not required for PPP2R5B-mediated regulation of ANPEP expression.

### PPP2R5B deficiency enhances HSF1 phosphorylation at Ser304/Ser308 and suppresses ANPEP transcription

**Fig 5:**
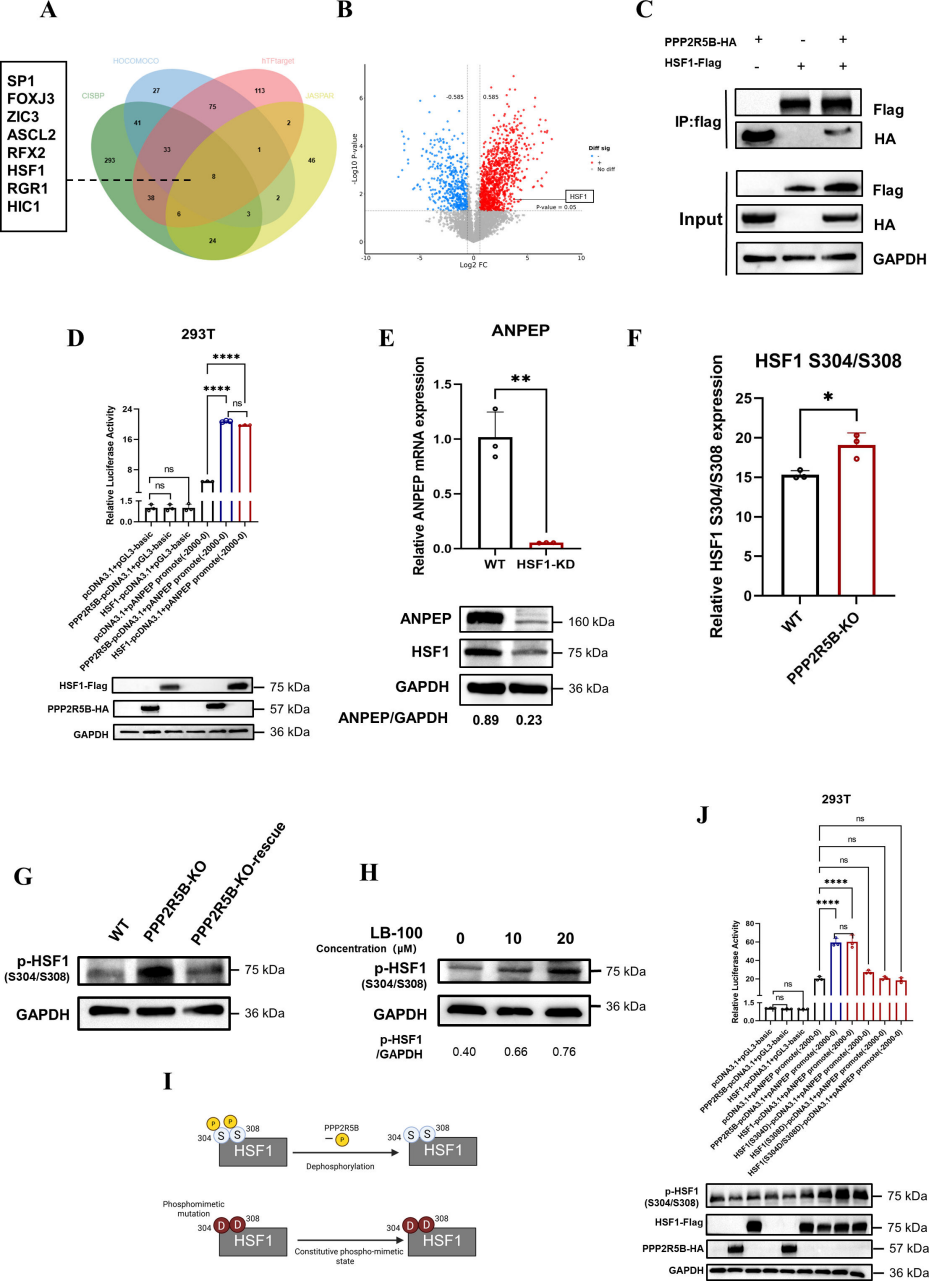
PPP2R5B deficiency enhances HSF1 phosphorylation at Ser304/Ser308 and suppresses ANPEP transcription. (**A**) Predicted transcription factors binding to the ANPEP promoter. (**B**) Volcano plot of differentially phosphorylated proteins identified by DIA-based phosphoproteomic analysis in WT and PPP2R5B KO PK-15 cells. (**C**) Co-immunoprecipitation (Co-IP) assays demonstrating the interaction between PPP2R5B and HSF1. (**D**) Dual-luciferase reporter assay assessing ANPEP promoter activity following overexpression of PPP2R5B or HSF1. (**E**) ANPEP mRNA and protein levels in HSF1 knockdown and WT cells. (**F**) Phosphoproteomic analysis showing increased phosphorylation of HSF1 at Ser304 and Ser308 in PPP2R5B KO cells compared with WT cells. (**G**) HSF1 phosphorylation at Ser304/Ser308 (the orthologous sites corresponding to human Ser303/Ser307) in WT, PPP2R5B KO, and PPP2R5B-rescued cells. Phosphorylation signals were detected using an anti-human phospho-HSF1 antibody that cross-reacts with the endogenous porcine protein. (**H**) Effects of PP2A inhibition (LB-100, 0-20 μM, 12 h) on HSF1 phosphorylation. (**I**) Schematic representation of the strategy used to generate phosphomimetic HSF1 mutants by substituting serine residues at positions 304 and/or 308 with aspartic acid. (**J**) Dual-luciferase reporter assays were performed to evaluate ANPEP promoter activity following overexpression of PPP2R5B, wild-type porcine HSF1, or phosphomimetic porcine HSF1 mutants (S304D, S308D, and S304/308D). Phosphorylation of the overexpressed porcine HSF1 constructs was detected using the same cross-reactive anti-human phospho-HSF1 antibody. Data are representative of at least three independent experiments. Statistical significance was determined by Student’s *t*-test or ANOVA as indicated. *P < 0.05; **P < 0.01; ***P < 0.001; ****P < 0.0001.

Computational prediction of the ANPEP promoter region (−2,000 to −1 bp) was performed using the JASPAR, hTFtarget, HOCOMOCO, and CISBP databases (Fig. 5A). DIA-based quantitative phosphoproteomic analysis identified 1,543 differentially phosphorylated sites, 70.27% of which were upregulated in PPP2R5B KO cells. The differential phosphoproteomic results are shown as a volcano plot (Fig. 5B). HSF1 was identified as a candidate transcription factor with predicted binding sites in the ANPEP promoter and markedly increased phosphorylation.

PPP2R5B functions as a substrate-recognition subunit of PP2A, conferring specificity to phosphatase activity. Co-immunoprecipitation assays revealed an interaction between PPP2R5B and HSF1 (Fig. 5C), suggesting that PPP2R5B-containing PP2A complexes may regulate HSF1 dephosphorylation.

To examine the role of HSF1 in regulating ANPEP transcription, dual-luciferase reporter assays were performed. Overexpression of either PPP2R5B or HSF1 significantly increased ANPEP promoter activity, with no significant difference between the two groups (Fig. 5D). Knockdown of HSF1 in PK-15 cells reduced ANPEP expression at both the mRNA and protein levels, as determined by RT-qPCR and Western blot analysis (Fig. 5E).

Analysis of the phosphoproteomic data revealed increased phosphorylation of porcine HSF1 at Ser304 and Ser308 in PPP2R5B knockout cells (Fig. 5F). Western blot analysis further validated these observations, demonstrating markedly elevated phosphorylation of HSF1 at Ser304/Ser308 in PPP2R5B KO cells, which was reversed upon PPP2R5B reconstitution (Fig. 5G). In WT cells, treatment with the PP2A inhibitor LB-100 resulted in a dose-dependent increase in HSF1 Ser304/308 phosphorylation, supporting a role for PPP2R5B-containing PP2A complexes in regulating HSF1 dephosphorylation (Fig. 5H).

To assess the transcriptional regulatory role of Ser304 and Ser308 phosphorylation in ANPEP expression, dual-luciferase reporter assays were performed. Phosphomimetic HSF1 mutants (HSF1-S304D, HSF1-S308D, and HSF1-S304/S308D) were generated by substituting serine residues with aspartic acid to mimic constitutive phosphorylation (Fig. 5I). All phosphomimetic mutants exhibited a pronounced reduction in ANPEP promoter activity, with luciferase levels comparable to those of the negative control (Fig. 5J).

**Fig 6:**
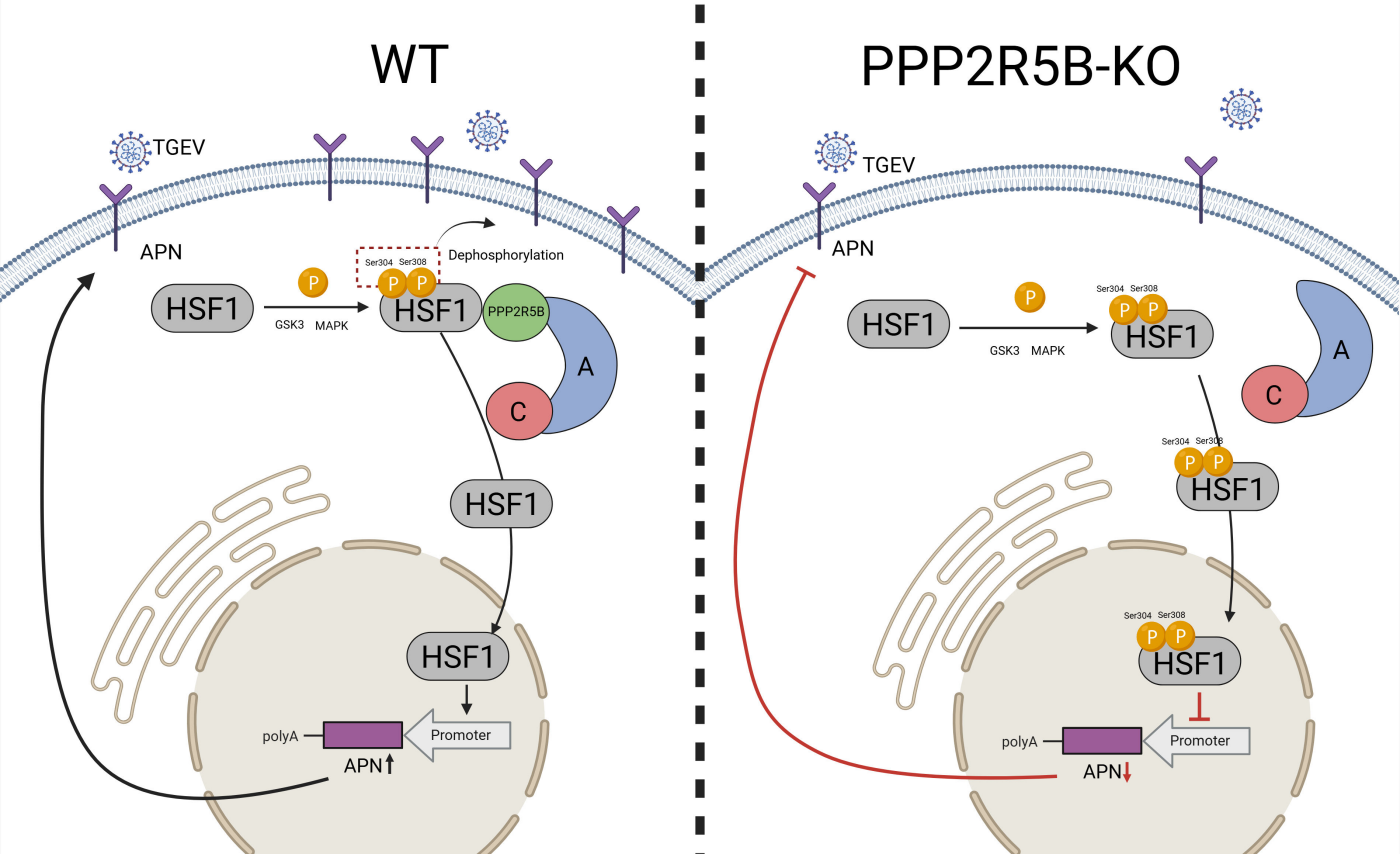
Model illustrating the PPP2R5B–HSF1–ANPEP regulatory axis during TGEV infection. TGEV initiates infection by binding to the host cell surface receptor aminopeptidase N (ANPEP/APN), thereby facilitating viral entry. Previous studies have shown that GSK3 and MAPK promote HSF1 phosphorylation at Ser304 and Ser308. In this study, PPP2R5B, as a regulatory subunit of the PP2A holoenzyme, promotes dephosphorylation of HSF1 at Ser304/Ser308, thereby enhancing its transcriptional activity and maintaining ANPEP expression. In PPP2R5B KO cells, loss of PPP2R5B-mediated dephosphorylation leads to HSF1 hyperphosphorylation, reduced transcriptional activity, and downregulation of ANPEP, ultimately impairing TGEV entry. This regulatory axis reveals a mechanism by which host phosphatase signaling modulates coronavirus infection.

Collectively, these results suggest that PPP2R5B promotes ANPEP transcription by maintaining HSF1 in a hypophosphorylated state at Ser304/Ser308, implicating HSF1 phosphorylation as a potential molecular switch linking PPP2R5B activity to ANPEP expression. Furthermore, validation of the reported upstream kinases of HSF1 (29–31) demonstrated that pharmacological inhibition of GSK3 (Fig. S2D) and MAPK (Fig. S2E) signaling in PPP2R5B KO PK-15 cells attenuated HSF1 hyperphosphorylation and restored ANPEP expression. Together, these findings define a PPP2R5B–HSF1–ANPEP regulatory axis (Fig. 6).

## DISCUSSION

This study provides the first evidence that PPP2R5B functions as a critical host factor during coronavirus infection. Building on our previous CRISPR screening results, we systematically elucidated the molecular mechanism by which PPP2R5B regulates TGEV infection. Our findings demonstrate that PPP2R5B does not act through canonical signaling pathways but instead it controls TGEV entry by modulating the phosphorylation status of HSF1 at Ser304/Ser308, thereby regulating the expression of ANPEP. These results expand our understanding of host dephosphorylation-dependent regulatory mechanisms in coronavirus infection and reveal a previously underappreciated host regulatory pathway.

Host cellular factors regulate the expression and posttranslational modification of the coronavirus receptor ANPEP through multilayered mechanisms. At the transcriptional level, several transcription factors, including ETS-2 (32), c-Maf (33), and DMP1 (34), have been shown to directly regulate ANPEP gene expression. More recently, the epigenetic regulator UHRF1 has been identified as a critical host restriction factor that limits ANPEP expression by maintaining DNA methylation at CpG sites within the ANPEP promoter region, thereby suppressing its basal transcriptional activity (35). Beyond transcriptional control, ANPEP is a highly N-glycosylated membrane glycoprotein, and its glycosylation status plays a decisive role in species-specific recognition by the spike proteins of alphacoronaviruses (36).

To date, most studies examining phosphorylation in coronavirus infection have focused predominantly on protein kinases, with CK2 (37, 38), CDKs (39, 40), p38-MAPK (41–43), and PKD (44) emerging as potential targets for antiviral intervention. In contrast, the role of phosphatases has received comparatively little attention. Protein phosphatase 2A (PP2A), one of the most abundant serine/threonine phosphatases in cells, has been implicated in the pathogenesis of multiple viral infections. PP2A can be directly targeted by viruses—for example, degradation of PP2A by HIV-1 Vif results in cell cycle arrest (45), or can act directly on viral proteins, such as dephosphorylation of VP30 during filovirus replication (46, 47). In addition, several viruses have been shown to hijack PP2A subunits to dampen host antiviral responses. For instance, the African swine fever virus protein p17 recruits the PP2A subunit PPP2R1A to promote dephosphorylation of IRF3 associated with STING, thereby suppressing type I interferon production (48). Despite these observations, the involvement of PP2A in coronavirus infection remains poorly characterized. Notably, PPP2R5B, a key regulatory subunit of PP2A, has been scarcely studied in the context of coronaviruses. Recent evidence indicates that increased PPP2R5B expression attenuates TBK1 and IRF3 phosphorylation, resulting in reduced interferon production and enhanced rotavirus infection (49), suggesting that PPP2R5B-mediated dephosphorylation may represent a broader viral immune evasion strategy.

An additional important finding of this study is that PPP2R5B regulates the dephosphorylation of HSF1 at Ser304/Ser308. Previous studies have reported that members of the PP2A-B55 family exert dual regulatory effects on HSF1 dephosphorylation through adaptor or regulatory proteins. Specifically, IER5 promotes dephosphorylation of HSF1 at Ser303, Ser307, and Ser320 (50, 51), thereby enhancing HSF1 transcriptional activity, whereas SERTAD1 interacts with HSF1 and facilitates dephosphorylation at Ser320 (52), resulting in reduced transcriptional activity. In contrast to these prior findings, our study provides the first evidence that a member of the PP2A-B56 family possesses the capacity to regulate HSF1 dephosphorylation. This observation expands the current understanding of HSF1 regulation and identifies PPP2R5B as a previously unrecognized node within the HSF1 regulatory network.

In the context of virus–host interactions, HSF1 plays a complex and critical role: on one hand, viruses can hijack HSF1 by promoting its phosphorylation, nuclear translocation, and induction of specific HSPs to facilitate viral replication (53); on the other hand, HSF1 acts as a host restriction factor that suppresses viral replication by inhibiting inflammatory pathways, such as NF-κB or modulating autophagy-related genes (54). As a transcription-associated regulatory factor, our study provides new evidence that HSF1 contributes to the regulation of coronavirus infection by modulating the transcriptional output of ANPEP. Our data indicate that phosphorylation of HSF1 at Ser304/Ser308 abolishes its ability to support ANPEP transcription. Ser304 and Ser308 are regulatory phosphorylation sites within the HSF1 regulatory domain, and phosphorylation at these residues has been associated with transcriptional repression, reduced protein stability, and attenuation of HSF1 activity (55, 56). At present, the precise mechanism by which HSF1 influences ANPEP expression remains unresolved. HSF1 may participate in ANPEP regulation by directly associating with regulatory elements within the ANPEP promoter, or alternatively, by functioning as a transcription-associated factor that cooperates with enhancer-associated complexes or other transcriptional regulators to indirectly modulate ANPEP transcription (57, 58). Further studies will be required to delineate the molecular basis of this regulatory process.

Although the PPP2R5B-HSF1-ANPEP axis identified in this study establishes a mechanistic link between PP2A-mediated dephosphorylation and TGEV infection in porcine cells, whether this regulatory mechanism extends to other ANPEP-dependent alphacoronaviruses remains to be investigated. Given that ANPEP serves as an entry receptor for multiple alphacoronaviruses across different species—including human HCoV-229E, porcine PEDV, and canine CCoV—it is reasonable to speculate that HSF1-mediated transcriptional regulation of ANPEP may represent a broadly relevant host defense mechanism. However, experimental validation using additional viruses and relevant cell systems is required to test this hypothesis. Furthermore, species-specific differences in ANPEP promoter architecture or HSF1 regulatory networks may influence the extent to which this axis is conserved.

To conclude, our work not only positions PPP2R5B as a critical host factor during TGEV infection but also reveals a previously uncharacterized regulatory axis involving HSF1 and ANPEP. These findings offer fresh insights into how coronaviruses interact with host cells and point to promising molecular targets for developing new antiviral therapies, thereby supporting ongoing efforts to counter emerging viral threats.

## MATERIALS AND METHODS

### Plasmid construction

Single-guide RNA (sgRNA) expression constructs were generated by ligating annealed sgRNA oligonucleotides into BbsI- or BsmBI-linearized lentiviral vectors (lenti-sgRNA-EGFP or lentiCRISPR-v2; NEB) according to established CRISPR cloning protocols. For complementation assays, synonymous substitutions were introduced into the sgRNA target regions of PPP2R5B and HSF1 to render them resistant to Cas9-mediated cleavage while preserving the encoded amino acid sequences. The resulting mutant fragments were subcloned into the pLVX-T2A-mCherry-Puro vector (Clontech) via XhoI and BamHI restriction sites. All plasmids were confirmed by Sanger sequencing, and primer information is provided in Table S2.

### Cell culture and viruses

PK-15 and HEK293T cells (Chinese Academy of Sciences Cell Bank) were cultured in Dulbecco’s modified Eagle’s medium (DMEM) containing 10% fetal bovine serum (FBS) and penicillin–streptomycin (100 U/mL and 100 μg/mL, respectively) at 37°C under 5% CO_2_ in a humidified atmosphere. Cells were routinely screened to ensure the absence of mycoplasma contamination. The TGEV WH-1 strain (GenBank accession no. HQ462571.1) was utilized throughout this study.

### Viral titers

Culture supernatants were collected at the specified time points after infection, and viral titers were quantified in PK-15 cells by calculating the 50% tissue culture infectious dose using the Reed-Muench method.

### Transfection and infection

Plasmid transfection was performed using jetPRIME reagent (Polyplus) in accordance with the manufacturer’s instructions. For infection assays, cells at approximately 80% confluence were exposed to TGEV for 1 h at 37°C, followed by washing with PBS and incubation in maintenance medium supplemented with 2% FBS. Infection levels were assessed by RT-qPCR and immunofluorescence analysis.

### Generation of candidate gene KO cell lines

CRISPR/Cas9-based gene disruption was conducted, as described previously. Lentiviral vectors harboring sgRNAs were generated and used to transduce PK-15 cells stably expressing Cas9. Transduced cells were enriched by fluorescence-activated cell sorting based on GFP expression, and single-cell-derived knockout clones were subsequently established.

### Western blotting and antibodies

Cells were lysed in lysis buffer supplemented with a protease inhibitor cocktail (Beyotime) on ice for 30 min. Cell lysates were clarified by centrifugation at 12,000 × *g* for 15 min at 4°C, boiled at 95°C for 10 min, and subjected to SDS-PAGE. Separated proteins were transferred onto PVDF membranes, which were blocked with 5% nonfat milk in TBST and incubated with primary antibodies at 4°C overnight. After washing, membranes were incubated with HRP-conjugated secondary antibodies for 1 h at room temperature. Protein signals were visualized using ECL Prime Western Blotting Detection Reagents (GE Healthcare). Primary antibodies used included: anti-TGEV N protein (1:1,000), anti-PPP2R5B (ABclonal, A19332, 1:5,000), anti-ANPEP (ABclonal, A11669, 1:3,000), anti-Phospho-AMPKα1-T183 + AMPKα2-T172 (ABclonal, AP1441, 1:5,000), anti-ERK1/2 (ABclonal, A4782, 1:5,000), anti-Non-phospho(Active)β-Catenin-S33/S37/T41 (ABclonal, A22180, 1:5,000), anti-Phospho-Akt (T308) (ABclonal, AP1259, 1:1,000), anti-AMPK Alpha (Proteintech, 10929-2-AP, 1:10,000), anti-Phospho-ERK1/2 (Thr202/Tyr204) (Proteintech, 80031-1-RR, 1:5,000), anti-Phospho-Akt (S473) (Genetex, GTX128414, 1:10,000), anti-AKT1/2/3 (Abmart, T55561, 1:2,000), anti-GAPDH (Proteintech, 60004-1-Ig, 1:10,000), anti-DYKDDDDK-tag (MBL, M185-3L, 1:10,000), anti-HA-tag (MBL, M185-3L, 1:1,000), and anti-Phospho-HSF1 (MCE, HY-P80822, 1:1,000).

Horseradish peroxidase (HRP)-conjugated secondary antibodies, including goat anti-rabbit IgG (Proteintech, RGAR001, 1:10,000) and goat anti-mouse IgG (Proteintech, RGARM005, 1:10,000), were applied, and the secondary antibodies were visualized using ECL Prime Western Blotting Detection Reagents (GE Healthcare, UK).

### RT-qPCR

Total RNA was isolated using TRIzol reagent (Invitrogen), and reverse transcription was carried out with the PrimeScript RT reagent kit (TaKaRa). Quantitative real-time PCR (qPCR) was performed using SYBR Green Master Mix (Bio-Rad) on a CFX96 real-time PCR system. Relative transcript levels were determined by the 2^−ΔΔCt^ method with β-actin serving as the internal reference. For absolute quantification, standard curves were established using plasmids containing the TGEV N gene.

### Immunofluorescence assay

TGEV N protein expression in candidate gene knockout and control cells was examined by immunofluorescence assay. Cells were infected with TGEV at the indicated multiplicities of infection (MOIs). At 24 hpi, cells were washed with PBS, fixed with 4% paraformaldehyde for 30 min at room temperature, and permeabilized with 0.3% Triton X-100 for 10 min. Following washing, cells were blocked with 5% bovine serum albumin (BSA) in PBS for 30 min.

Cells were incubated with a rabbit polyclonal antibody against TGEV N protein (in-house generated) for 2 h at 37°C, followed by thorough washing with PBS. Cells were then incubated with Alexa Fluor 594-conjugated secondary antibodies for 1 h at 37°C in the dark. After additional washes, nuclei were counterstained with DAPI for 2 min at room temperature. Fluorescence images were acquired using an EVOS FL Auto fluorescence microscope (Thermo Fisher Scientific).

### Cell viability assay

Cell viability was evaluated using an MTS-based assay (Abcam, ab197010) in accordance with the manufacturer’s protocol. Metabolic activity was quantified by measuring absorbance at 490 nm.

### Virion attachment and internalization assay

Cells were incubated with virus at 4°C for 1 h to allow attachment, as described previously (59). For endocytosis assays, cultures were subsequently shifted to 37°C and incubated for 1 h, followed by acid washing to remove non-internalized virions. Intracellular viral RNA levels were then quantified by RT-qPCR.

### RNA sequencing and transcriptome analysis

For high-throughput RNA sequencing, RNA libraries were generated for different experimental groups, comprising PPP2R5B-KO cells (PPP2R5B-KO-MOCK and PPP2R5B-KO-TGEV) and WT cells (WT-MOCK and WT-TGEV), each performed in triplicate. Poly(A)+ RNA isolation, library construction, and sequencing were conducted by a commercial provider. All samples underwent quality assessment with FastQC, and reads were trimmed using Trimmomatic. The resulting high-quality reads were mapped to the Sus scrofa reference genome (v11.1) with HISAT2 (v2.2.1). Gene expression levels were quantified as fragments per kilobase of exon per million mapped reads (FPKM) for each unigene using FeatureCounts (v2.0.8). Differentially expressed genes (DEGs) were identified with DESeq2 (v1.46.0), applying a significance threshold of *P* ≤ 0.05 and an absolute fold change ≥2. Pairwise comparisons of DEGs were subsequently analyzed for pathway enrichment using KEGG and GO analyses implemented in the ClusterProfiler package (v4.14.3).

### Inhibitor treatment assay

Cells were treated with specific inhibitors, including LB-100 (MCE, HY-15431), Compound C (MCE, HY-13418A), AICAR (MCE, HY-13417), and Capivasertib (MCE, HY-15431), prepared as stock solutions and diluted to working concentrations prior to use.

### Prediction of transcription factors

The promoter region of ANPEP was analyzed using JASPAR (https://jaspar.elixir.no/), hTFtarget (https://guolab.wchscu.cn/hTFtarget/#!/), HOCOMOCO (https://hocomoco11.autosome.org/), and CISBP (https://cisbp.ccbr.utoronto.ca/) databases to identify potential binding transcription factors. The integrated results were visualized in a Venn diagram to pinpoint transcription factors common across these databases.

### Phosphoproteomic analysis

Protein extraction, enzymatic digestion, phosphopeptide enrichment, and LC-MS/MS analysis were performed by a commercial vendor according to established protocols. Raw data were analyzed using FragPipe, and peptide quantification was conducted with IonQuant. Differential phosphorylation analysis and downstream functional annotation were performed using standard bioinformatics workflows.

### Co-immunoprecipitation assay

HEK-293T cells were grown in six-well plates and then co-transfected with pCAGGS-PPP2R5B-HA along with pcDNA3.1-HSF1-Flag for 24 h. After transfection, the cells were rinsed three times with ice-cold PBS and lysed in Cell Lysis Buffer suitable for both Western blotting and immunoprecipitation (Beyotime). The resulting lysates were cleared by centrifugation at 12,000 rpm for 10 min, and the clarified supernatants were mixed with 10 μL of anti-Flag magnetic beads (MCE, HY-K0207). This mixture was incubated for 6–8 h at 4°C. The beads were then collected and washed three times with ice-cold PBS, each wash followed by centrifugation at 2,500 rpm for 5 min at 4°C. Finally, the immunoprecipitated proteins were subjected to Western blotting analysis.

### Dual-luciferase reporter assay

A 2,000-bp fragment upstream of the ANPEP gene was amplified and inserted into the pGL3-Basic vector upstream of the luciferase reporter. HEK-293T cells were seeded in 24-well plates and cultured for 24 h prior to transfection. Cells were then cotransfected with the luciferase reporter construct, pRL-TK plasmid, pcDNA3.1-PPP2R5B, pcDNA3.1-HSF1, pcDNA3.1-HSF1 mutants, or corresponding control plasmids using JetPRIME reagent (PolyPlus) according to the manufacturer’s protocol. At 24 h posttransfection, luciferase activities were determined using a Dual-Luciferase Reporter Assay Kit (Vazyme, DL101-01). Firefly luciferase activity was normalized to Renilla luciferase activity to control for transfection efficiency.

### Statistical analysis

Statistical significance was assessed using GraphPad Prism 8.0 software. For data analysis, two-tailed unpaired *t*-tests were used for comparisons between two groups, while comparisons involving more than two groups were performed using ANOVA, followed by appropriate post-hoc tests. Unless otherwise stated, data represent the mean ± standard deviation of at least three independent experiments.

## Data Availability

The data that support the findings of this study are openly available in this article and are available from the corresponding author after request.

## SUPPLEMENTAL MATERIAL

Supplemental figures jvi.00166-26-s0001.docx Fig. S1 and S2.

Table S1 jvi.00166-26-s0002.xlsx Differentially phosphorylated sites identified between PK-15-Cas9 cells (WT) and PPP2R5B KO cells by phosphoproteomic analysis.

Table S2 jvi.00166-26-s0003.xlsx Primer list.

Table S3 jvi.00166-26-s0004.xlsx Predicted transcription factors binding to the ANPEP promoter region.

ASM does not own the copyrights to Supplemental Material that may be linked to, or accessed through, an article. The authors have granted ASM a non-exclusive, world-wide license to publish the Supplemental Material files. Please contact the corresponding author directly for reuse.
