# Supplementary material for: PPP2R5B regulates ANPEP expression and TGEV entry via dephosphorylation of HSF1 at Ser304/Ser308
Source: J Virol. 2026 Jun 11;100(7):e00166-26. doi: 10.1128/jvi.00166-26 (PMC13387003; doi:10.1128/jvi.00166-26)
Supplement: Supplemental figures — Fig. S1 and S2. [file jvi.00166-26-s0001.docx]

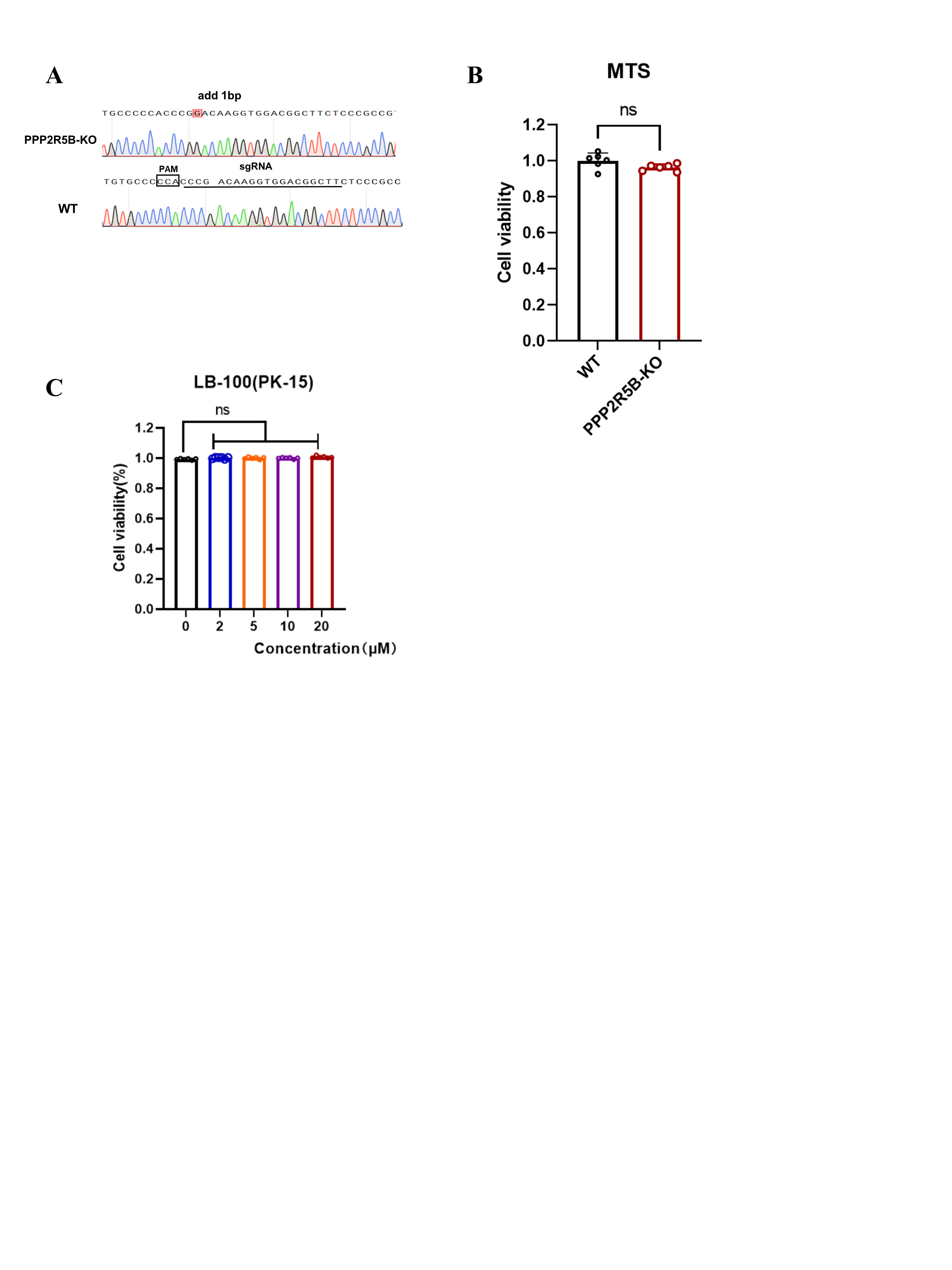


**Fig. S1. Construction and validation of PPP2R5B cell lines.** (A) Alignment of nucleotide sequences from PPP2R5B knockout cells and wild-type cells. The sgRNA and PAM sequences are shown in the wild-type sequence, with inserted nucleotides highlighted in red. (B) PPP2R5B KO and WT PK-15 cells were seeded into 96-well plates, and cell viability was measured by MTS assay at 24 hpi. (C)PK-15 cells were treated with or without LB-100 for 48 h, and cell viability was verified by MTS assay


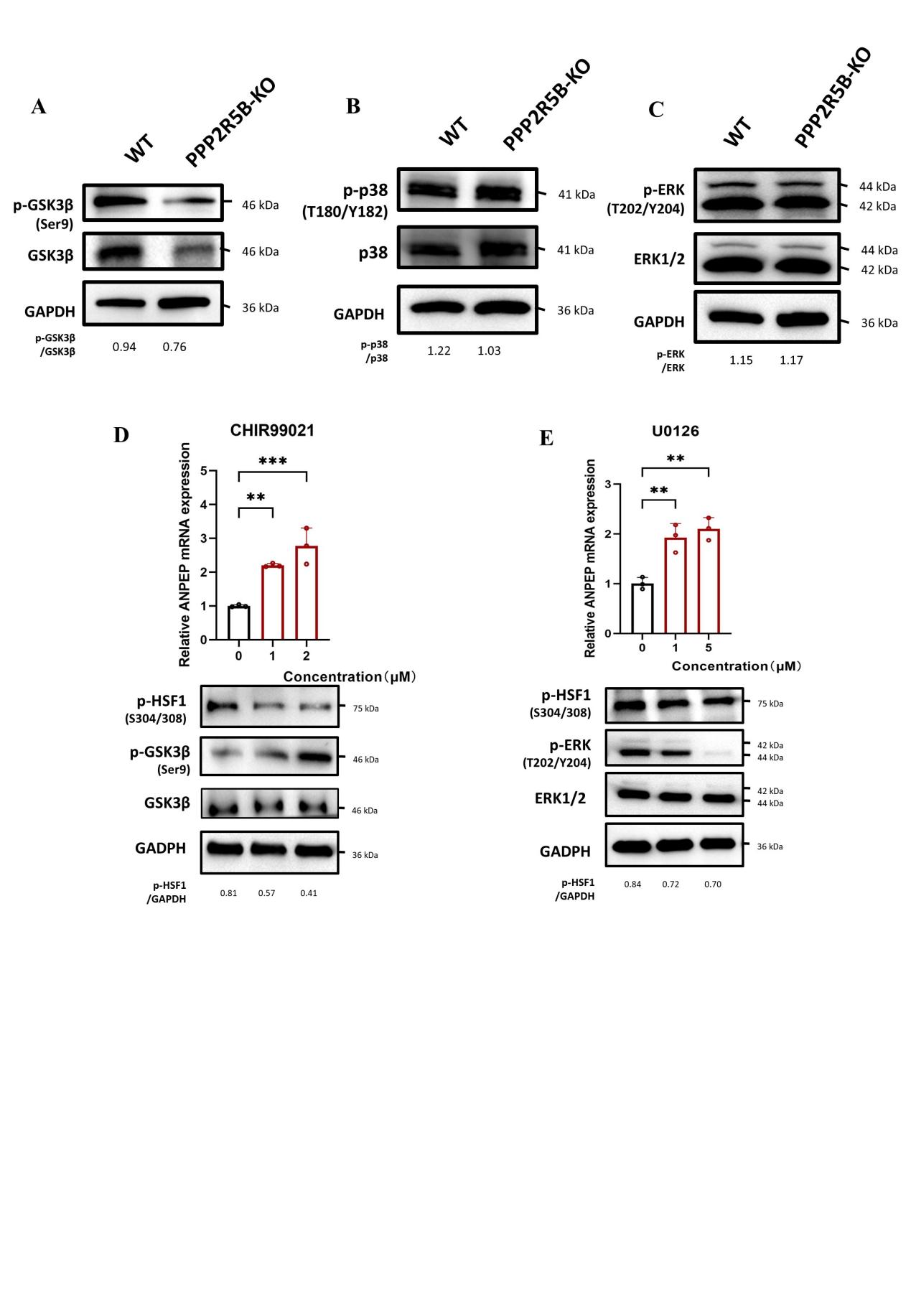
**Fig. S2. Validation of upstream kinases regulating the PPP2R5B–HSF1–ANPEP pathway** (A) Phosphorylation and total protein levels of GSK3β were analyzed by Western blot in WT and PPP2R5B KO cells. Increased phosphorylation at Ser9 indicates reduced GSK3β kinase activity. (B) Phosphorylation and total protein levels of p38 were analyzed by Western blot in WT and PPP2R5B KO cells. (C) Phosphorylation and total protein levels of ERK were analyzed by Western blot in WT and PPP2R5B KO cells. (D) PPP2R5B KO cells were treated with the GSK3β inhibitor CHIR99021 (0, 1, and 2 μM)for 6 h. HSF1 phosphorylation and total and phosphorylated GSK3β levels were analyzed by Western blot, and ANPEP mRNA expression was measured by RT-qPCR. (E) PPP2R5B KO cells were treated with the MEK1/2 inhibitor U0126 (0, 1, and 5 μM) for 6 h. HSF1 phosphorylation and total and phosphorylated ERK1/2 levels were analyzed by Western blot, and ANPEP mRNA expression was measured by RT-qPCR.
